# Media-Dependent Effects in Heart Rate Variability Biofeedback: A Systematic Review of Extended Reality and Screen-Based Training Approaches

**DOI:** 10.1089/jmedxr.2024.0060

**Published:** 2025-05-09

**Authors:** Nathan Miner, Karmen Gill, Casper Harteveld, Christopher Bono, Darshan H. Mehta

**Affiliations:** ^1^ Department of Orthopaedics, Massachusetts General Hospital, Boston, Massachusetts, USA.; ^2^ College of Arts, Media and Design, Northeastern University , Boston, Massachusetts, USA.; ^3^ Department of Biology, Union College, Schenectady, New York, USA.; ^4^ Department of Orthopaedic Surgery, Harvard Medical School, Mass General Brigham, Boston, Massachusetts, USA.; ^5^ Benson-Henry Institute for Mind Body Medicine, Massachusetts General Hospital, Boston, Massachusetts, USA.; ^6^ Osher Center for Integrative Health, Harvard Medical School and Brigham and Women’s Hospital, Boston, Massachusetts, USA.

**Keywords:** systematic review, health applications, heart rate variability, biofeedback, wearable sensors, extended reality, virtual reality, augmented reality, mixed reality

## Abstract

Heart rate variability (HRV) is an indicator of autonomic nervous system function and cardiovascular health. Through biofeedback (BF), individuals can increase their HRV, which is ideal, by using audiovisual cues to monitor and adjust their breathing. HRV-BF training has demonstrated effectiveness in alleviating stress and anxiety, enhancing cardiac autonomic function, and supporting psychological treatments for conditions, such as depression and chronic pain. Despite their benefits, traditional HRV-BF approaches are limited by the need for frequent clinical visits, specialized equipment, and trained personnel, limiting accessibility and scalability. Extended reality (XR) technologies, including virtual reality, augmented reality, and mixed reality, offer a promising alternative by creating immersive, interactive environments that can deliver HRV-BF without traditional constraints. This review examines the available literature on reported differences in user experience (UX), HRV metrics, psychological outcomes, and BF performance, which are documented by research comparing HRV-BF using XR media to traditional screen-based methods. We searched PubMed, PsycINFO, Embase, Cochrane, Science Direct, ACM, and IEEE databases. Our search yielded 382 results. Four studies met our review criteria. Using HRV-BF research reporting guidelines, we extracted and summarized study objectives, design strategies, hypotheses, and related outcomes to evaluate the evidence for media-dependent effects. Findings indicate that both XR and screen-based implementations of HRV-BF positively affect HRV physiology. Furthermore, XR-based HRV-BF implementations showed more pronounced effects on outcomes for psychological and UX variables. These studies suggest that XR media can offer advantages in terms of user engagement and psychological benefits (e.g., stress relief, relaxation) over traditional screen-based modalities. Improvements in UX and psychological outcomes stem from the relaxing, immersive contexts created in XR, supporting user motivation and attentional control during XR-based HRV-BF training. However, design-based inquiries and longitudinal studies are needed to understand the role of design and the potential of the specific mechanisms contributing to these results.

## Introduction

Measurement of variations in heart rate over time (i.e., heart rate variability [HRV]) offers a view into the underlying state of the autonomic nervous system (ANS). HRV indices are measured in the time and frequency domains and through non-linear dynamics. HRV measures can help assess adaptability to change, where diminished variability suggests greater vulnerability to psychological and physical stresses and disease.^1^ Low HRV is correlated with various health conditions (e.g., hypertension, diabetes, and anxiety disorders). HRV indices have been explored to identify autonomic dysfunction, assess stress levels, and monitor cardiovascular health. HRV biofeedback (HRV-BF) training has also been developed and applied as a therapy to increase HRV in conditions for which lower HRV is a known correlate.^2,3^ Research on the best methods for studying and applying HRV-BF training is still emerging.

HRV is closely linked to breathing, with heart rate increasing during inhalation and decreasing during exhalation, a phenomenon known as respiratory sinus arrhythmia (RSA). This relationship allows targeted breathing practices, such as resonant frequency (RF) and slow-paced breathing (sPB), to effectively enhance HRV. These breathing practices optimize the natural oscillations between heart rate and breathing by syncing with the body’s baroreflex response to respiration-related blood pressure changes. This alternately compresses and stretches the R–R interval (RRI) of the heart rate to enhance respiration efficiency and support the nervous system’s homeostatic balance. By controlling the depth and pace of breathing during breathing exercises, the resulting compression and stretching of the heart rate maximizes the variations in RRI, providing an exercise for the heart and nervous system. BF, a technique that involves monitoring a user’s biological signals and providing real-time feedback, enables individuals to gain awareness and control over physiological functions, thereby improving or altering the underlying biological mechanisms.^4^ HRV-BF typically uses paced breathing techniques to guide individuals through breathing exercises while providing feedback on the HRV metrics of interest, usually via visual or auditory cues. Standard metrics for HRV-BF include cardiac coherence, RSA, standard deviation of normal-to-normal (NN) intervals (SDNN), root mean square of successive differences (RMSSD), low-frequency power (LF), high-frequency power (HF), the LF/HF ratio, among others.^5^ Research into the effects of HRV-BF is typically designed around a collection of HRV metrics, and manipulations of breathing frequencies during interventions are typical).

There has been growing interest in wearable sensors and extended reality (XR) media as new tools in BF interventions and research, as these systems can allow measurement and training outside traditional clinical contexts. In particular, XR-based interventions have shown promise in motivating and engaging users to perform therapeutic tasks.^10,11^ Studies comparing traditional media to XR-based media have observed positive media-dependent effects.^12^ The current review examines the evidence exploring media-related effects in XR-based HRV-BF training.

## Methods

**FIG. 1.**
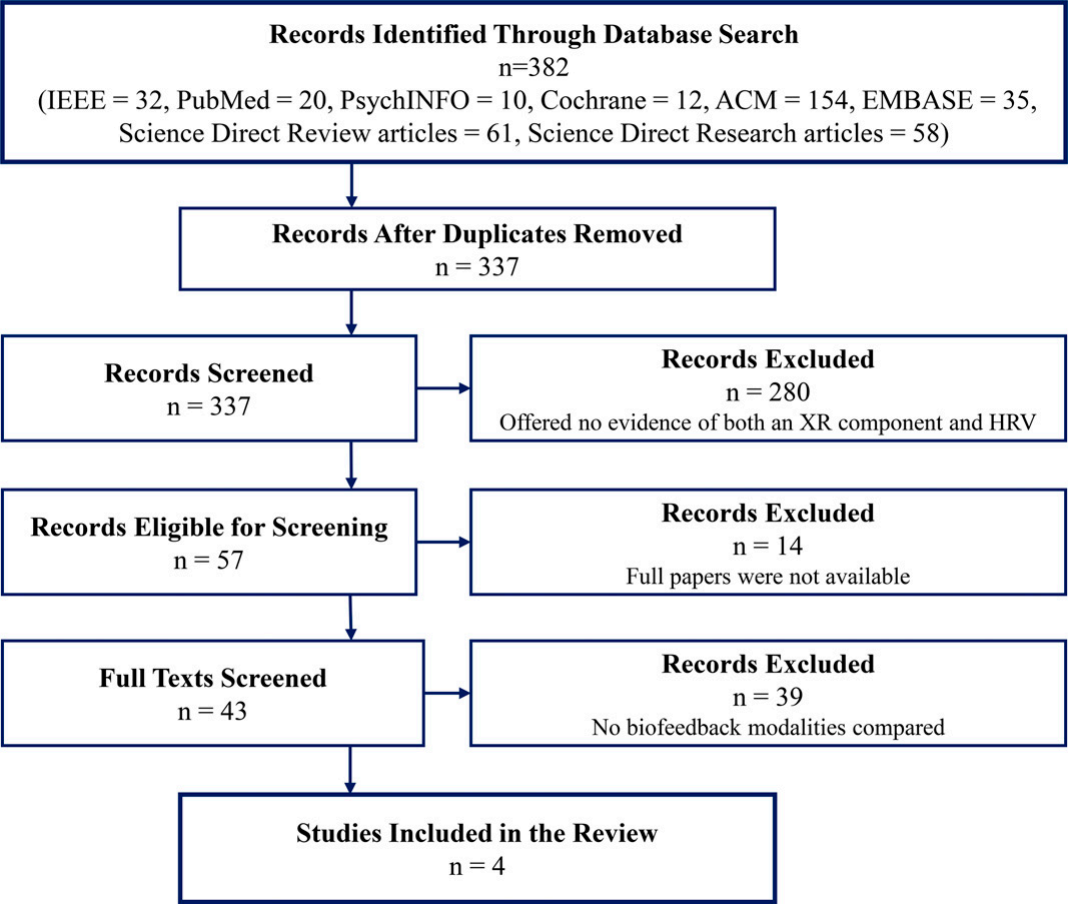
Flowchart detailing the review selection process.

The primary question we asked was “How can XR media alter or enhance responses to HRV-BF in health-related treatments or evaluations compared to traditional interventions?” Our review included searches performed on January 11, 2024, from PubMed, PsycINFO, Embase, Cochrane, Science Direct, ACM, and IEEE databases, returning four studies that met our review criteria from 382 studies (Fig. 1). The condition and domain studied were limited to HRV-BF and XR media for BF delivery. There was no exclusion from the population sample. The search string (“extended reality” OR “virtual reality” OR “augmented reality” OR “mixed reality”) AND (“HRV” OR “heart rate variability” OR “heart rate” OR “variability”) AND (“Biofeedback, Psychology” OR “biofeedback”) was used to find articles. The language of the articles was limited to English. There were no restrictions on study type [e.g., (Randomized Controlled Trial) RCT, cohort studies].

### Study selection

Two reviewers independently applied eligibility criteria and selected studies for inclusion. Each reviewer screened records for inclusion independently using Rayyan.ai to examine titles, abstracts, and metadata for HRV and XR media components. We limit XR to implementations involving a head-mounted display (HMD), explicitly excluding other forms, such as (Cave Automatic Virtual Environment) CAVE systems. Researchers were blind to each other’s decisions during screening, and disagreements between reviewers regarding inclusion were resolved through discussion.

The first round of screening excluded studies that did not mention an XR media component and the use of HRV. Additional studies were excluded if a full article could not be found (e.g., conference demos, posters, abstract-only proceedings, or clinical trials without an associated article). The remaining studies were imported into Zotero for further review and filtered for inclusion following a two-step process: (1) the article objectives were confirmed to include the use of XR media, HRV, and BF in their analysis and (2) the articles directly compared XR-based HRV-BF with a traditional HRV-BF method.

### Appraisal, data extraction, and management

We use the guidelines provided by Lalanza et al.^13^ as an assessment tool, applying their checklist for improving the methodological rigor and reporting in HRV-BF studies as a method of comparison and appraisal in our review (see Appendix).

We examined the studies to create descriptive tables detailing the study sample, results, and author conclusions. Outcome measures for studies varied to include standard physiological metrics for HRV parameters in time and frequency domains, such as RMSSD, SDNN, NN50, PNN50, LF and HF, and LF/HF ratio, as well as psychometric and UX variables. Psychometric variables relied on custom self-report and standardized questionnaires for attention, stress, anxiety, depression, and pain. UX data were also extracted. Details regarding effect sizes and *p*-values from the statistical tests of each study were extracted to create a comprehensive overview of each study. Differences in the designs, implementations, and techniques, along with the type and duration of measures, did not allow quantitative comparisons. Instead, we summarize and compare each study qualitatively.

## Results

Thirty-nine of the 43 full articles screened did not include a direct comparison of XR media to traditional screen-based HRV-BF methods. Where comparisons were made in this larger body of research, only four provided a comparable BF component in both conditions. Subsequent sections refer to the reviewed studies by the last name of the first author: Prabhu,^6^ Blum,^7^ Rockstroh,^8^ Weibel.^9^ The studies focused on psychophysiological outcomes, particularly the potential to reduce stress and anxiety (Table 1).

**Table 1.** Study Design, Population, Setting and Sessions, Results, and Conclusions

Study	Study design	Study population	Study setting and sessions	Results	Conclusions
Prabhu et al., 2024^6^	Explore the design and performance of a VR-based HRV-BF in a nature-themed environment compared with a traditional screen-based HRV-BF for use in mitigating surgical pain and anxiety.	*n* = 27, TKA patients divided into three groups (passive control [*n* = 9], traditional screen-based HRV-BF [*n* = 9], and VR-based HRV-BF [*n* = 9]), 66.3 ± 8.2 years.	Hospital pre- and post-surgical setting with 3-min baseline measurement, 5-min practice, and two HRV-BF sessions, one pre-surgery and one post-surgery.	Both methods increased HRV parameters (RMSSD, LF/HF ratio) and decreased respiration rate compared to controls. STAI and VAS measures of pain and anxiety were reduced. Usability scores were similar. Presence was higher for VR (except for realism).	Screen-based and VR-based methods are similarly beneficial for mitigating pain and anxiety and increasing HRV metrics post-surgery compared to untreated controls.
Blum et al., 2019^7^	Compare VR HRV-BF using a nature-themed environment with traditional screen-based HRV-BF for biofeedback performance, attention, relaxation, and relaxation self-efficacy.	*n* = 60, healthy employees in two groups (standard-BF [*n* = 29] and VR-BF [*n* = 31]), 31 females, 29 males, 33.5 ± 9.4 years.	Laboratory experiment (double-blind, randomized, controlled, between-subjects) with one 10-min HRV-BF session.	Both methods improved cardiac coherence and cardiac vagal tone. VR improved relaxation self-efficacy, reduced mind wandering, increased focus on the present moment, and conserved attentional resources compared to the standard method.	VR-based implementation is more beneficial than traditional media for relaxation self-efficacy, reducing mind wandering, increasing focus, and conserving attentional resources during HRV-BF training.
Rockstroh et al., 2019^8^	To explore the feasibility and potential advantages of VR-based HRV-BF using a nature-themed environment versus traditional screen-based HRV-BF versus non-treated control.	*n* = 68, healthy participants in three groups (control [*n* = 22], standard-BF [*n* = 22], VR-BF [*n* = 24]), 41 females, 27 males, 22.9 ± 4.0 years.	Laboratory experiment (randomized, controlled, between-subjects) with one 10-min HRV-BF session.	Both methods increased HRV parameters (SDNN, coherence ratio, LF/HF ratio) compared to controls; VR showed higher motivation, better attentional focus, and reduced distraction compared to traditional HRV-BF.	VR-based HRV-BF is feasible and offers motivational and attentional advantages over traditional methods.
Weibel et al., 2023^9^	Compare the effects of VR-based HRV-BF to traditional screen-based HRV-BF, and compare VR-based sPB without BF to traditional screen-based sPB without BF on stress and HRV.	*n* = 107, healthy participants divided into four groups (traditional screen-based HRV-BF [*n* = 25], VR-based HRV-BF [*n* = 25], traditional screen-based sPB [*n* = 29], and VR-based sPB [*n* = 28]), 48 females, 59 males, 22.52 ± 3.33 years.	Laboratory experiment: 3-min practice, two 10-min HRV-BF or sPB training sessions.	All methods showed beneficial psychological and cardiac effects relative to stress management. Resonant frequency breathing showed greater effects for HRV (SDNN, cardiac coherence, and low-frequency power).	All conditions significantly decreased perceived stress and improved mood, calmness, wakefulness, and relaxation. HMDs provided higher immersion and interface quality ratings than desktop screens. However, screens had higher ratings for facilitating conditions, particularly for sPB.

HMD, head-mounted display; HRV-BF, heart rate variability biofeedback; LF/HF, low-frequency to high-frequency ratio; RMSSD, root mean square of successive differences; SDNN, standard deviation of normal-to-normal intervals; sPB, slow-paced breathing; STAI, State–Trait Anxiety Inventory; TKA, total knee arthroplasty; VAS, Visual Analog Scale; VR, virtual reality.

Despite method variations, the studies agreed that immersive nature-themed virtual reality (VR) environments would enhance usability and UX and yield better psychophysiological outcomes than traditional screen-based HRV-BF. Prabhu et al.^6^ explored VR-based HRV-BF for use in mitigating surgical pain and anxiety. Blum et al.^7^ compared VR with traditional screens for BF performance, attention, relaxation, and relaxation self-efficacy. Rockstroh et al.^8^ explored the feasibility of using VR-based HRV-BF for relaxation. Weibel et al.^9^ compared the effects of VR and traditional screens using sPB and RF breathing on stress management and HRV. The following sections detail the similarities and differences between the methods, theories, technologies, and media design strategies used in the studies.

### Study methods

Prabhu et al.^6^ evaluated the efficacy of VR-based HRV-BF in reducing surgical pain and anxiety among total knee arthroplasty (TKA) patients. They hypothesized that VR-based HRV-BF and a 2D video with HRV-BF would significantly mitigate pre- and post-operative pain and anxiety, expecting VR to show stronger effects. The study also hypothesized that older adults undergoing TKA would accept the HRV-BF interventions well. Cardiac activity was monitored using a Biopac® MP160 system with a BioNomadix® BN-RSPEC wireless transmitter-receiver. Data were preprocessed to remove artifacts and analyzed using mixed-effects models and analysis of covariance, controlling for pre-intervention scores. Presence and usability were assessed using the iGroup Presence Questionnaire and the System Usability Scale. According to the authors, both VR and 2D video with HRV-BF were found to significantly reduce pre- and post-operative pain and anxiety compared to the control group.

Blum et al.^7^ hypothesized that VR-based HRV-BF would be comparable to screen-based methods in improving cardiac coherence and vagal tone. In addition, they postulated that VR-based HRV-BF would reduce perceived stress during a subsequent stressor task, increase relaxation self-efficacy, reduce mind wandering, enhance focus on the present moment, and conserve attentional resources better than traditional HRV-BF. The study included measures of relaxation self-efficacy (State–Trait Anxiety Inventory), mind wandering (Cognitive Interference Questionnaire [CIQ]), focus on the present moment (State Mindfulness Scale [SMS]), and attentional resources using a Stroop task.

In the Blum study, the immersive VR environment provided additional benefits regarding stress buffering. Both BF methods increased relaxation and significantly increased cardiac coherence and vagal tone. VR-BF showed a greater ability to buffer stress in a subsequent stressor task, supporting the author’s hypothesis that VR enhances the stress-shielding effects of HRV-BF. VR also led to greater increases in relaxation self-efficacy than screens, suggesting that the immersive feedback in the VR environment boosted participants’ confidence in their ability to relax. They further reported less mind wandering and greater focus on the present moment, as indicated by higher scores on the SMS and lower scores on the CIQ. Participants in the VR condition also had better reaction times on the Stroop task, suggesting conservation of attentional resources.

Rockstroh et al.^8^ hypothesized that a VR-based HRV-BF would be as effective as a traditional HRV-BF in increasing short-term HRV, resulting in higher motivation and better maintenance of attentional focus. The goal was to reach and maintain a state of heart coherence. VR and screen-based HRV-BF successfully increased HRV parameters, demonstrating the feasibility of VR-based HRV-BF. The authors find the VR condition associated with higher motivation, better attentional focus, and greater enjoyment than screen-based HRV-BF. Participants in the VR condition reported higher intention to use and recommend the system and perceived time to pass more quickly during the session. The authors suggest that findings demonstrate VR-based HRV-BF as a feasible and effective method for stress management, offering additional benefits in terms of motivation and attentional focus, highlighting the potential of VR to enhance UX and engagement in HRV-BF training.

Weibel et al.^9^ made five hypotheses postulating that HRV-BF and sPB would significantly improve psychological and cardiac measures (e.g., stress, mood, wakefulness, and HRV variables) over screen-based sPB. The study evaluated the baseline characteristics of the participants to ensure a balance across groups using the Perceived Stress Scale-10, the Depression Anxiety Stress Scale-21, and the World Health Organization Five Well-Being Index. In addition, general health perception was assessed using the Short Form Health Survey—General Health Perception Subscale, whereas the Basic Psychological Needs Satisfaction and Frustration Scale measured satisfaction and frustration of basic psychological needs. The Immersive Tendency Questionnaire was also used to assess participants’ tendency to become immersed in an environment.^14^ The training session was divided into three blocks, with physiological and psychological measures taken before, during, and after the training. This design allowed for the comparison of the BF technique (HRV-BF vs. sPB) and the type of display technology (HMD vs. desktop screen). Testing blocks for BF conditions included a 6-min recording to determine the RF following the protocol of Sakakibara et al. and two 10-min testing blocks for all conditions.^15^

Data were processed in real-time with a custom Python script. Both conditions increased perceived relaxation, calmness, good mood, and wakefulness during the training session. However, there was no significant difference between them, with both conditions significantly increasing HRV features. UX measures were generally positive for all conditions, with higher involvement reported for HRV-BF and better interface quality and immersion adaptation for HMDs.

### Theories

Each study offered common theoretical justifications for their design and use of VR, such as heightened immersion, greater presence, and more captivating visuals compared to traditional 2D screens.^16,17^ Rationales for using BF were also similar, citing evidence for better health outcomes in subjects with higher HRV and established evidence for HRV-BF to increase HRV metrics and improve cardiac coherence while lowering stress.^1,18–20^ Because stress and anxiety affect the ANS and interfere with the body’s ability to heal, interventions reducing stress and anxiety, such as mindfulness-based stress reduction (MBSR), are now well-established as complementary care in integrative medicine.^21,22^ MBSR teaches participants to focus on the present moment non-judgmentally, enhancing emotional resilience and promoting well-being.^22^ It is also used in health care as a complementary approach to support mental and physical health. The effectiveness of HRV-BF in psychological studies of anxiety has also been established,^23^ and each study referenced aspects of these in the foundational arguments of their work (Table 2).

**Table 2.** Technology and Data Processing, Design Theories, Experience Design, and Biofeedback Design

Study	Technology and data processing	Design theories	Experience design	Biofeedback design
Prabhu et al., 2024^6^	Unity Game Engine, HTC Vive VR headset, 2880 × 1600 pixel resolution, 110° field of view, 90 Hz refresh rate, Biopac® MP160 system for heart rate monitoring and signal processing, over-the-ear headphones, breathing rate at 0.1 Hz (6 BPM).	Stress reduction theory, gate control theory, multiple resource theory	The virtual environment included a beach scene with a blue sky, natural wave movement, a sun in the front view, and palm trees with green grass in the side views. The soundscape featured sea waves and wind.	A foggy beach environment transitioned to a clear one when participants maintained their optimal breathing rate for 1 min. If they strayed for over 30 s, fog reappeared. Sine waves acted as the biofeedback component, whereas fog served as a gamification element to incentivize adherence.
Blum et al., 2019^7^	Unity Game Engine, Oculus Rift CV1, 2160 × 1200 pixel resolution, 110° field of view, 90 Hz refresh rate, Polar H7 chest strap, custom Python application with BLE on a Windows 10 computer, over-the-ear headphones, breathing rate at 0.1 Hz (6 BPM).	Neurovisceral integration model, polyvagal theory, vagal tank theory, Kaplan’s attention restoration theory	The virtual environment included a serene beach landscape at sunset with palm trees, rocks, a campfire, a twilight sky, and ocean views with sailing boats.	Breathing was paced with an audio track of a person breathing. Visual and auditory feedbacks were integrated into the environment. Positive feedback reduced cloud coverage, revealing a clear starry sky while lamps and a campfire lit up. Negative feedback restored cloud coverage and dimmed the lights.
Rockstroh et al., 2019^8^	Unity Game Engine, Oculus Rift CV1, 2160 × 1200 pixel resolution, 110° field of view, 90 Hz refresh rate, Polar H7 chest strap, custom Python application with BLE on a Windows 10 computer, over-the-ear headphones, breathing rate at 0.1 Hz (6 BPM).	Kaplan’s attention restoration theory	The virtual environment was lit softly with lanterns, torches, and a campfire. The beach scene framed the space as private, with an ambient ocean, wind, and instrumental music soundscape.	Breathing was paced with an audio track of a person breathing. Positive feedback cleared clouds, lit campfires and torches, turned on ambient lighting, and set sailboats moving. These changes were reflected in softer wind and wave sounds. Negative feedback reversed all effects. Seven lamps signaled accumulated feedback scores.
Weibel et al., 2023^9^	Unity Game Engine, Oculus Quest, 2880 × 1600 pixel resolution, 100° field of view, 72 Hz refresh rate, Polar H10 chest strap, custom Python application using Polar SDK, audio delivered through VR headset, breathing rate at RF and 0.1 Hz (6 BPM).	Cardiac coherence model, resonance frequency model, neurovisceral integration model, cognitive affective model of immersive learning	The environment depicted a mountainous landscape, with participants sitting on a tree trunk overlooking a grassy meadow. Surrounding elements included trees, bushes, boulders, and distant mountain peaks. The sun was either fixed or rising gradually, and the soundscape included bird songs and a flowing creek.	Breathing was paced with a semi-transparent white cylinder and a disc moving up and down. Positive feedback included a brighter and more colorful environment; increased HRV was signaled by flower growth in a meadow, a rising sun, and an intensified natural soundscape.

BLE, Bluetooth Low Energy; BPM, breaths per minute; HRV, heart rate variability; RF, resonant frequency; SDK, software development kit.

Three of the four studies cited Kaplan’s attention restoration theory (ART),^24^ which suggests that people’s attentional resources become depleted, reducing their ability to cope with stress.^6–8^ According to ART, attentional resources can be replenished through relaxation in expansive natural settings, with specific qualities that contribute to their restorative effects.^24,25^ The Blum study^7^ delved deeper into the theories underlying HRV-BF, referencing the neurovisceral integration model,^26^ polyvagal theory,^27^ and vagal tank theory^28^ to collectively explain the psychophysiological interdependence and the mind–body dynamics involved in social and emotional self-regulation.^7^ The Prabhu study^6^ supported the goals of their work by citing the stress reduction theory,^29^ which posits that natural and nature-themed environments can effectively reduce stress. Prabhu also discussed the gate control theory^30^ and multiple resource theory,^31^ proposing that VR can help relieve pain and anxiety by diverting attention away from these sensations and occupying attentional capacities with immersive interactions.^6^

The Weibel study^9^ included such theoretical frameworks as the cardiac coherence model,^32^ the resonance frequency model,^33^ and the neurovisceral integration model.^26^ Weibel et al. use these theories to explain how BF’s mechanisms positively affect physiology and further explain the utilities of VR for enhancing engagement, learning, and experience, citing the cognitive affective model of immersive learning (CAMIL).^34^ The CAMIL theory describes how qualities of immersion enhanced by a user’s sense of control affect feelings of presence and agency in VR. The theory suggests these enhancements, in turn, influence cognitive and affective factors such as interest, motivation, self-efficacy, embodiment, cognitive load, and self-regulation, promoting knowledge acquisition and learning transfer. Although these theories provide a rich conceptual foundation for the studies, the assumptions inherent in them were not explicitly tested.

### Technology and data

All four studies used the Unity Game Engine^35^ to create custom virtual environments made of 3D models (Table 2). The Prabhu study employed an HTC Vive VR headset,^36^ whereas the other studies used Meta/Oculus devices.^37^ The Weibel study delivered audio directly through the VR headset, whereas the other studies used over-the-ear headphones. The screen-based BF was presented on desktop computers with large monitors (24 in.). The Prabhu study used Biopac sensors and software^38^ for heart rate monitoring and analysis, whereas the other studies relied on Polar H10 and Polar H7 devices.^39^

Each project required a data collection and signal processing strategy for accessing and filtering the raw RRI/inter-beat intervals (IBI) data. Prabhu et al.^6^ used the Biopac MP160 data acquisition system. The applications using Polar devices each created a customized solution using Python. The Blum and Rockstroh studies employed a custom “man-in-the-middle” application on a Windows 10 computer via Bluetooth Low Energy for their Polar H7 chest strap. They used Python for the processing and analysis functions.^7,8^ Following a similar approach, Weibel et al.^9^ used Python’s “hrv” and “scipy” libraries to create a custom application for the Polar H10 chest strap data using the Polar software development kit.

Breathing at a rate of 0.1 Hz (i.e., 6 BPM) is the commonly used frequency for sPB, and all four studies used this rate for at least one condition. This breathing rate has been shown to amplify HRV LF power, helping to balance parasympathetic and sympathetic nervous system activity.^1^ Slow breathing at RF, typically around 0.1 Hz but unique to everyone, creates a resonant condition in the nervous system from the frequency oscillations of the heart and respiration rate.^33^ Only Weibel et al.^9^ used RF in their study. sPB, especially at the RF, can stimulate the baroreflex response, reduce blood pressure, and maximize increases in HRV, making both sPB and RF breathing effective tools for modulating physiological states, promoting relaxation, and improving cardiovascular health.^33^

### Media design

VR was the only XR modality used in the four studies. Each study presented an approach framed by the combined logic of using VR for immersion and engagement and HRV-BF for efficacy in treating stress and anxiety. The studies positioned their work by arguing that VR media and HRV-BF could work together to enhance and amplify each other’s positive effects in interventions. Although virtual nature-themed environments are not technically natural, the reviewed studies argue that nature simulated in XR can evoke similar sensory experiences and restorative outcomes, according to the principles of Kaplan’s ART.^24^ These studies suggest that immersive, nature-themed VR environments can fulfill ART’s four criteria, “fascinating” users through the “extent” of the immersive experiences, fostering a sense of “being away” from the routines of daily life in ways that are “compatible” with users’ desires and goals.^24^

The studies detailed their rationale for the experience design aesthetics and BF representations. Each project used a nature theme to design the virtual environment and used environment changes to contextualize and represent the BF. Blum^7^ and Rockstroh^8^ explored the feasibility and efficacy of a tropical beach-themed virtual environment to promote relaxation and reduce stress. Prabhu^6^ also employed a beach-themed natural environment with a custom BF design and used this representation for both the VR and screen-based conditions. The Weibel study^9^ developed an alpine meadow environment with wildflowers and mountains for stress management. Prabhu et al.^6^ designed a fog effect that clouded and obscured the clarity of an otherwise sunny beach scene to provide negative feedback when the breathing pace was not aligned with the objective rate. The fog cleared as participants achieved optimal breathing rates (Table 2).

Although none of the designs used by the reviewed studies had been experimentally validated as BF methods before these studies, the Rockstroh study investigated their system’s feasibility, and the same team of authors, led by Blum, assessed their system’s efficacy for stress reduction. The design of this environment included a virtual beach and sunset, with positive feedback represented by environmental changes, such as lighting campfires, turning on lanterns, and clearing the sky. An audio-based breathing pacer and visual BF were embedded in the audiovisual design of the VR environment. Both studies compared their VR-based method to a screen-based experience modeled on traditional approaches using minimalistic graphical representations for BF.^7,8^

The Weibel study used the sun’s progress across the sky and flowers blooming in the meadows as positive feedback.^9^ The virtual environment depicted a serene, mountainous landscape viewed from a grassy meadow surrounded by trees and bushes, with mountain peaks in the distance. They included audio elements such as bird songs and a flowing creek to enhance immersion and relaxation and to accompany visual effects, like the rising sun. Changes to the virtual environment represented BF: the rising sun, moving flowers, and an intensifying soundscape. A translucent white cylinder with a disk moving up and down was placed in the middle of the meadow to pace participants’ breathing. The Weibel study used the same virtual environment on the screen- and VR-based conditions.

### Synthesis of results

The studies collectively demonstrated that XR and traditional screen-based HRV-BF interventions significantly improved HRV parameters without observing significant differences between the two modalities. However, compared to control groups, significant differences were found for SDNN, RMSSD, coherence, and LF/HF ratio in both XR and screen-based conditions, with RF breathing conditions in the study of Weibel et al. showing the greatest improvements. Despite similarities between media modalities for physiological measures, XR-based HRV-BF was associated with differences in psychological and UX measures compared to traditional methods. Beneficial effects related to stress management were also observed for treatment conditions across the studies. XR-based HRV-BF received higher immersion and interface quality ratings, whereas traditional screens were rated higher for facilitating conditions.^9^

Regardless of the technology, all HRV-BF conditions reduced perceived stress and improved mood, calmness, wakefulness, and relaxation. However, the studies collectively suggest that XR-based delivery can offer superior usability outcomes by enhancing the user’s engagement and focus during HRV-BF training. The XR-based interventions showed greater relaxation self-efficacy, reduced mind wandering, increased focus on the present moment, and conservation of attentional resources compared to standard HRV-BF. These enhancements indicate that XR can effectively augment and support the psychological aspects of HRV-BF training. Although not reporting adverse events from the interactions, the authors emphasized the importance of optimizing designs to avoid inducing cybersickness and tuning experiences to the specific needs of the target populations. Interest in and usability of XR were high even in the older population of Prabhu et al.^6^ In addition, Weibel et al.^9^ highlighted that while XR experiences were engaging, users reported a stronger sense of competence with more familiar, traditional technologies.

## Discussion

We find greater evidence for improvements in psychological and UX metrics due to the use of XR media than for alterations or enhancements of physiological measures of HRV compared to traditional screens. However, these media-dependent effects suggest that improvements in HRV metrics may emerge from multisession longitudinal studies due to the training improvements offered by XR’s attentional, motivational, and engagement-related advantages. Both XR and screen-based HRV-BF methods showed substantial improvements over controls, and these similarities demonstrate the feasibility and potential of using XR for BF research to increase accessibility and deepen engagement with BF training.

Here, we discuss the traditional approaches and clinical applications of HRV and BF training and the rationale for applying XR media in this domain. We consider the efficacy of XR demonstrated here, highlight opportunities for methodological improvement in future studies, and detail the current research gaps related to the need for longitudinal studies and engagement of clinically relevant populations.

### HRV and BF training

**FIG. 2.**
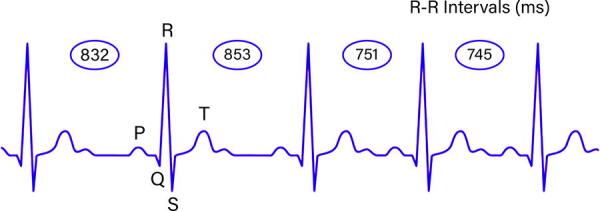
Heart rate signal from an electrocardiogram sensor showing the PQRST sequence of the heart’s electrical activity and used for the derivation of heart rate variability from measures of the distance in milliseconds between consecutive R peaks, known as R–R interval, inter-beat interval, and normal-to-normal.

Twenty-nine indices derived from HRV analysis contribute to a nuanced understanding of the autonomic function and are valuable in clinical and research contexts (Table 3). Monitoring and comparing these over time for an individual can expose interrelated dimensions of psychological, physiological, and autonomic health.^28,41^ HRV naturally fluctuates daily in response to physical and mental rest, stress, activity, diet, and sleep cycles and decreases with age. Monitoring heart rates for 24 h and environment controls are recommended for time domain analysis, whereas 5-min intervals are recommended for frequency domain variables.^40,42^ HRV can be derived from electrocardiogram and photoplethysmography signals and is calculated by analyzing changes in time intervals between consecutive R-peaks, known as RRIs, IBI, and normal-to-normal (Fig. 2).

**Table 3.** Heart Rate Variability Indices and Domains of Analysis^40^

Analysis domain	Sub-domain	Acronym	Description
Time domain	Deviation-based approach	SDNN	Standard deviation of NN intervals
		SDANN	Standard deviation of average NN intervals for each 5-min segment
		SDNN index	Mean of the standard deviations of NN intervals in 5 min segments
	Difference-based approach	SSDD	Standard deviation of successive NN interval differences
		RMSSD	Root mean square of successive NN interval differences
		pNN20	Proportion of successive NN interval differences larger than 20 ms
		pNN50	Proportion of successive NN interval differences larger than 50 ms
	Geometric approach	HTI	Integral of the density of the NN interval histogram divided by its height
		TINN	Baseline width of the RR interval histogram
Frequency domain	Absolute power	ULF	Power spectrum in the frequency range of ≤0.003 Hz
		VLF	Power spectrum in the frequency range of 0.0033–0.04 Hz
		LF	Power spectrum in the frequency range of 0.04–0.15 Hz
		HF	Power spectrum in the frequency range of 0.15–0.4 Hz
	Normalized/relative power	LnHF	Natural logarithm of HF
		HFn	Normalized HF
		LFn	Normalized LF
		LF/HF	Ratio of LF to HF power
Time–frequency domain	Linear approach	STFT	Power spectrum estimation using short-term Fourier transform
		WT	Power spectrum estimation using Wavelet transform
	Quadratic approach	WVD	Power spectrum estimation using Wigner–Ville distribution
		SWVD	Power spectrum estimation using smoothed Wigner–Ville distribution
Non-linear domain	Poincaré plot	SD1	Poincaré plot standard deviation perpendicular to the line of identity
		SD2	Poincaré plot standard deviation along the line of identity
		SD1/SD2	Ratio of SD1 to SD2
	Entropy	ApEn	Estimation of complexity using approximate entropy
		SampEn	Estimation of complexity using sample entropy
		MSE	Estimation of complexity using multiscale entropy
	Fractal dimensions	DFA	Estimation of signal fluctuations using detrended fluctuation analysis
		CD	Estimation of minimum number of variables to define a dynamic model

NN, normal-to-normal interval; RR, R–R interval.

Studies have shown that sPB can improve patients’ subjective well-being, addressing symptoms of stress, anxiety, depression, pain, and sleep quality.^43,44^ In addition, paced breathing at RF (i.e., the individualized breathing rate that optimizes HRV by creating a state of resonant synchrony in the nervous system) can significantly enhance the effectiveness of HRV-BF training.^33,45^ sPB, usually around six breaths per minute (6 BPM [0.1 Hz]) combined with HRV-BF, resulted in better emotional control and higher HRV metrics than sPB alone.^44^ sPB and RF breathing have also been shown to improve physiological coherence between heart rate and respiratory rhythms, leading to better outcomes in stress reduction and overall autonomic regulation.^45^

Clinically, HRV has been explored for diagnostics and therapy. Studies have examined HRV-BF for medical and mental health disorders, including asthma, chronic pain, heart failure, hypertension, depression, and post-traumatic stress disorder.^46^ Reviews of HRV-BF have provided an overview of the potential efficacy in improving health outcomes by enhancing autonomic regulation, especially in cases related to stress and anxiety conditions.^18,19^ HRV-BF has been shown to enhance emotional and autonomic regulation, thereby reducing symptoms associated with these conditions.^1^ In addition, HRV-BF can complement clinical interventions and treatments, including cognitive-behavioral therapy, stress management programs, performance enhancement for athletes and individuals in high-stress occupations, and cardiac rehabilitation.^47^

Traditionally, HRV-BF training is conducted in clinical settings and administered under the guidance of a trained therapist or clinician, who can interpret the BF data and provide personalized coaching and adjustments to the training protocol. Advancements in wearable technology and mobile applications can make HRV-BF more accessible outside traditional contexts and provide insight into the changes in the various domains of HRV throughout the day and night.^48^ Wearable sensors, such as the ActiGraph LEAP,^49^ can provide continuous long-term HRV monitoring in the home environment. This provides clinicians with important contextual information for subjects’ psychological and physiological data while allowing patients to manage their health at home.

XR and other mobile health (mHealth) applications have also been explored to engage patients in home-based care, especially for gathering ecological momentary assessments and delivering guided training or other interventions, including HRV-BF training.^50^ VR, in particular, has been explored as a mHealth tool for delivering BF training due to its advantages in teaching and guiding users, controlling attention, and engaging people in performing specific tasks while offering new data collection methods for behavioral and physiological responses.^51^ The combined opportunities of mHealth applications, XR, and wearable sensors are now being explored for their potential to advance BF training, mind–body interventions, and longitudinal HRV research.^10,11,52^

## The Rationale for Using XR Technologies in HRV-BF Training

XR technologies are emerging as strategic health and wellness tools, leveraging immersive and customizable virtual environments, gamified interactions, and real-time feedback for therapeutic interventions. As the hardware and software for XR media rapidly evolve, new opportunities for XR-based treatment protocols are also emerging. As interest in XR-based BF continues to grow, so does the research investigating the mechanisms of action and clinical applications for BF therapies, and it is crucial to understand how and to what extent these mechanisms are bound to the UX, design, and media used for BF delivery.

Two prior reviews on BF and XR motivated the current inquiry on XR and HRV-BF. In the review of Kothgassner et al.,^53^ the authors examined the use of XR to enhance BF treatments of anxiety, showing it to be similarly effective to, but not significantly more effective than, traditional modalities. Similarly, in the scoping review of Lüddecke and Felnhofer,^54^ the authors sought to understand how XR-based BF implementations might overcome challenges faced in traditional BF, such as poor motivation, attentional focus, and a lack of skill transfer from training to everyday life. We conducted the current review to understand how the immersive experiences designed for XR have been used to facilitate HRV-BF training and to what degree they enhance training efficacy or deepen user engagement compared to other methods.

Our search returned four articles directly comparing the differences between XR-based HRV-BF and traditional screen-based BF methods. These articles focused on the general effectiveness of immersive virtual environments in improving UXs and HRV-BF performance. Common among the articles was the use of the media for relaxation with stress and anxiety management. One study also explored the potential of XR-based HRV-BF to mitigate post-operative surgical pain.^6^ We synthesize the evidence to identify the benefits and challenges of XR when directly compared to non-immersive HRV-BF methods. We examine the use cases and outcomes established to date and highlight the knowledge gaps for future research. We aim to understand if and how XR-based HRV-BF implementations can be engineered to improve HRV training and interventions.

### A foundation established: The feasibility of XR-based HRV-BF

The history of XR-based BF research has been pioneering, exploratory, and foundational. Still, recent reviews show that XR-based BF research is maturing in quantity, quality, and sophistication of studies.^53,54^ Technical advancements in XR media from the hardware and software industries have lowered device costs and improved development conditions.^55^ Studies adjacent to those in our review, such as BreathCoach,^50^ show the technical advancement supporting the gamification of BF and the use of mobile technologies to make RSA-BF training accessible. The studies in this review, especially that of Weibel et al.,^9^ demonstrate that the foundation for XR media delivery of HRV-BF is established and can successfully be used in large trials.

Other recent advancements are documented in the larger, clinically relevant populations in two studies found by our search.^56,57^ Although excluded from our review due to their lack of controlled media comparisons, Weerdmeester et al.^57^ engineered DEEP, a mobile VR BF game to reduce anxiety. They examined its efficacy in a clinical trial with 112 participants. Their findings indicated that DEEP users reported higher levels of self-efficacy and engagement with the VR system; however, there was no significant reduction in anxiety compared to the control group using the smartphone-guided breathing app.^57^ In addition, Kim et al.^56^ tested a VR system for stress reduction on a relevant sample of 74 high-stress individuals. They noted that users’ experience of simply relaxing in VR without BF was found to have comparable effects to that of a traditional screen-based BF intervention, illustrating the power of immersive media. Although no significant difference in subjective stress reduction was found between the VR experience and BF, physiological measures, such as electromyography and HRV indices, showed a difference: VR more effectively increased HRV metric NN50.^56^ The studies of Weerdmeester et al.^57^ and Kim et al.^56^ offer further support for this review’s findings, showing XR’s potential to motivate users in therapeutic engagements with BF training.

With a technical foundation established for XR-based HRV-BF interventions, research can now advance to address clinical needs. For this advancement, a better understanding of the role of the XR experience and the mechanisms behind the efficacy of feedback designs needs to be better understood. Yet, an XR-based HRV-BF gold standard does not exist for new research to reference. For the projects in this review, attending to the technical integrations and experience design of the XR application development was a principal research undertaking. Although the foundation is established, further work can be done to provide technical resources, reducing the complexity of HRV-BF application development, data collection, and analysis. Without reference to a common standard, our ability to examine the role of design in the delivery of HRV-BF training will remain limited. Methodological improvements are also needed to standardize the comparability of variables in HRV-BF interventions, as suggested by Lalanza et al.^13^

### Methodological advancement of XR-Based HRV-BF research

We use the guidelines provided by Lalanza et al.^13^ for insight into improving the reporting of methodological details in HRV-BF studies. In our review, each study used different protocols and physiological measures, and missing details in reporting made it impossible to compare findings quantitatively. This section further considers how XR media studies can help advance HRV-BF research.

### Analysis, sample size, and population

The studies in this review did not report registration or pre-registration, but this rigor could have strengthened their evidence. For example, the Prabhu study^6^ reported changes to their sample size due to study limitations but did not detail how these changes affected their analysis. Furthermore, they did not differentiate between pain and anxiety using the LF/HF ratio and self-reported measures to assess them as a single metric. Nor did they account for the effects of anesthesia on their sample’s physiological measures. The Prabhu study was the only investigation in this review to target a relevant sample population of older TKA patients in a pre- and post-operative setting. They were also the only study with a respiration belt sensor for breathing frequency verification but did not report any analyses or findings related to the respiration data. The results of the Prabhu study suggest a positive response to the intervention. Still, the sample and number of sessions were not large or well-controlled enough to produce unequivocal evidence.

Although guidelines for best practices have been established for data analysis,^40^ translating these into XR-based BF applications requires additional considerations. Even a subject’s body position is important for studies to be comparable. For example, three of the four studies in this review used RMSSD as an HRV metric. However, for the patients in the protocol of Prabhu et al.,^6^ the HRV measurements of the reclining group were taken in a hospital bed at 120°. In contrast, the other groups were taken while seated upright. Because the heart’s physiology changes relative to body position, HRV measurements taken while reclining and seated differ fundamentally. Although the same HRV variable was used across these studies, the measurements are not directly comparable due to these positional differences.

### Improving study design with technology

For consistent measurement and analysis of HRV, it is recommended to use dedicated software such as Kubios,^58^ which allows for visual inspection of the data and provides automated processes for identifying, correcting, or removing noise and ectopic beats. In contrast, custom Python solutions operating in real time to generate BF cannot offer the same level of data analysis. Since even a single R-peak error can significantly distort HRV metrics, protocols must include robust methods for handling signal noise in data processing.^40^ XR applications can be designed to help control the conditions of experiments and gather further data about training sessions, such as body and eye movements, and the timing of actions taken by users in the virtual environment. Movement data could also be used in UX studies to understand an application’s usability and effectiveness for analysis within and between subjects.

Each of the studies in this review could have reported more details on the conditions of their interventions. Lalanza et al.^13^ recommend protocols to report on environmental factors, such as participants’ positions, time of day, ambient light, and room temperature, which can also affect the comparability of HRV metrics. Factors, such as age, sex, height, weight, and body mass index, are also implicated in HRV measures. Blum et al.^7^ explain exclusion criteria (e.g., cardiovascular disease, psychological disorders, epilepsy, severely impaired balance, smoking, and regular medication use) and their protocol’s stipulation to participants to refrain from caffeine, smoking, and exercise on the day of the experiment, as these can alter metabolic processes and put a strain on the heart.

Although similar details were described in the other studies, no study explained how to control for participant adherence to these conditions. The HRV-BF protocol of Lehrer et al.^23^ further suggests limiting strenuous exercise and alcohol consumption on the day before an intervention. The sensitivity of HRV measures to environmental and individual factors increases the challenge of comparing HRV-BF groups and studies. Given these complexities, designing protocols and methodologies that support multiple BF sessions with longitudinal data collection poses additional challenges.

Advancing research using XR for BF necessitates standards for common comparisons between studies. None of the design implementations documented in this review had been previously examined or validated. Although they were each found effective and comparable to traditional screen-based methods, interrogating the role and effectiveness of the designs themselves would only be possible with a standard reference and consistently used protocol for HRV measures and analysis. Although we find the interactive potential of XR-based media to be effective, the specifics of the environment and BF design and the execution of the immersive content are expected to influence the quality and effectiveness of an intervention. Therefore, to advance XR-based HRV-BF research, design and human–computer interaction research studies are needed to compare intervention outcomes alongside UXs.

### A need for longitudinal studies

HRV-BF therapies typically involve multiple sessions.^23^ Physiological responses to HRV interventions, while emergent and measurable during a single BF training session, may also result in HRV changes outside the observational window of a single-session study. For example, meditation and deep breathing exercises typically show immediate physiological changes to HR but are also used to improve sleep. With a longitudinal study design, effects outside of standard observational windows can be examined. For example, stress lowers HRV, and meditation and breathing exercises can increase HRV and lower stress. Because of this, meditation and breathing practices are commonly used to help people restore a healthy balance between sleep and waking states, enhancing the body’s ability to cope with and recover from stress. However, these effects are not necessarily immediate. Continuous HRV monitoring is recommended to understand how meditation and breathing practices during the day affect HRV both at the moment and later that day or night.^40^

Although the reviewed studies noted the potential for media-related effects to produce significant physiological differences for XR-based delivery, they suggest multiple sessions and longitudinal data would be needed for these observations.^7,9^ A wearable device for continuous measurement would also be required, and an accompanying mHealth application for wearable data collection could support longitudinal investigations of HRV-BF training, therapies, and interventions.

Although the technologies are now available to build XR-based HRV-BF training systems for longitudinal studies, and RF breathing has been shown to have distinct advantages on HRV, the best method of determining a user’s RF and assessing the stability of breathing frequency over time still requires more research. Determining an individual’s RF is a step that is not always taken since additional analysis is needed. In our review, Weibel et al. were the only ones to include RF detection in their protocol and found that this method produced the best results.^9^ Fisher and Lehrer^59^ developed an RF breathing detection protocol using a sliding window for analysis and a pacer incrementally slowing breathing between 6.75 and 4.5 BPM over 15 min. Sakakibara et al.^15^ tested an alternative method requiring 5 min of breathing at 15 BPM (0.25 Hz). They produced comparable results but noted that more research is needed to investigate their method with larger and more diverse populations. Accessible longitudinal XR-based sPB applications could also be used to study RF breathing at a scale to advance our understanding of its bearing on HRV.

### Limitations of review

The scope of our review included only those four studies that explicitly compared XR and screen-based HRV-BF. Through this, we gain a focused, in-depth analysis of the XR experiences and their HRV-BF outcomes. However, we miss out on the diversity of design strategies and the broader range of efficacies found in the wider literature, and this limited scope reduces the generalizability of our findings. In addition, the variability in study designs within our review, including differences in technological implementations and outcome measures, did not allow a quantitative comparative analysis, limiting our conclusions. Additional reviews incorporating a more comprehensive range of studies are still needed to examine the mechanisms of action and the role of design in XR-based implementations of HRV-BF interventions.

## Conclusions

Our review shows that research in this domain has yet to explore large, demographically diverse, clinically relevant populations or engage participants in longitudinal studies of XR-based HRV-BF training. Although the BF therapies that our reviewed studies were based on have proven beneficial for clinical conditions, and evidence suggests potential for therapeutic effects for other conditions involving ANS dysregulation, questions remain regarding how to deliver HRV-BF to these populations. Questions also remain as to which HRV variables should be used and how to control for and collect the data needed to understand the outcomes of such interventions. Specifically, future studies must effectively gather the necessary HRV metrics while controlling for, or at least documenting, the individual human and environmental factors implicated in measures of HRV. The success of prior work motivating and engaging users with XR media may help advance our understanding of the mechanisms underlying HRV-BF therapies. Like the HRV metrics, these mechanisms may be enmeshed in the circadian rhythms of life and habit and only be visible after longer-term training engagements. The potential differences in experience design and motivation shown for XR media in these studies could make longitudinal studies of BF training more accessible and engaging for the durations needed to see the expected benefits of HRV-BF therapies.

## Abbreviations Used

AR: Augmented Reality
BF: Biofeedback
HRV: Heart rate variability
MR: Mixed reality
VR: Virtual reality
XR: Extended reality

## Appendix

**Appendix Table A1.Checklist and Reporting Details for Reviewed Studies:**

Methodological guidelines for HRV-BF research (Lalanza et al., 2023)^9^	Prabhu et al., 2024^10^	Blum et al., 2019^11^	Rockstroh et al., 2019^12^	Weibel et al., 2023^13^
(1) Pre-registration or registration	None	None	None	None
(2) Initial sample (final sample)	*n* = 30 (*n* = 26)	(*n* = 60)	(*n* = 68)	*n* = 115 (*n* = 107)
Power analysis: calculation of the minimum sample size to obtain an appropriate effect size.	Included	N/A	N/A	Included
Biological sex: number of women and men per group	Initial 23 females, 7 males Final N/A	31 females, 29 males	41 females, 27 males	Final 48 females, 59 males
Age: mean age for each group	66.3 ± 8.2 years	33.5 ± 9.4 years	22.9 ± 4.0 years	22.52 ± 3.33 years
Condition: e.g., students, cardiovascular patients, or those suffering from obesity	TKA patients	Healthy adults	Healthy adults	Healthy adults
Weight (optional): mean weight for each group	N/A	N/A	N/A	N/A
Height (optional): mean height for each group	N/A	N/A	N/A	N/A
(3) Allocation				
Recruitment of participants/patients/clients to the study	Hospital patients	Personal and professional networks	Social media and online database	Online university recruitment
Compensation for participating in the study (payment, credits, holidays, etc.)	$10	N/A	N/A	$50
Inclusion and exclusion criteria were assessed and explained	Detailed	Detailed	N/A	Detailed
Awareness or suspicion of participants of the experimental group assignment	Not blinded	Not blinded	Not blinded	Not blinded
Random (optional): assignation	Randomized	Randomized	Randomized	Randomized
(4) Missing participants and missing data				
Initial sample: once the participants have agreed to participate or come to the first session	*n* = 30	*n* = 60	*n* = 68	*n* = 115
Final sample: participants included for the data analysis	*n* = 26	*n* = 60	*n* = 68	*n* = 107
Causes for losing participants/patients (optionally use a diagram)	Withdrawal	N/A	N/A	N/A
Missing data for the statistical analysis	N/A	N/A	N/A	Technical difficulties
(5) Breathing protocol				
Type of HRV-BF intervention based on the category of breathing technique. The type of intervention determines which items need to be completed	Slow paced	Slow paced	Diaphragmatic	Resonant Frequency and Slow Paced
Resonance frequency (RF) detection (optional): details about the establishment of the individual RF. This item also includes when and how many times the RF was detected across the entire HRV-BF intervention	N/A	N/A	N/A	One 6-min measure following Sakakibara et al., 2020^15^
Mean of the RF (optional) for the experimental group	N/A	N/A	N/A	5.44 BPM
“Preset or fixed” RF (optional): for the “preset-pace” or slow-breathing intervention	6 BPM (0.1 Hz)	6 BPM (0.1 Hz)	6 BPM (0.1 Hz)	6 BPM (0.1 Hz)
Control of breathing rate (optional): for intervention with a collective or individual resonance frequency, e.g., use of a respirometer	Biopac® BioNomadix® BNRESP respiration band	N/A	N/A	N/A
Inhalation, holding, and exhalation seconds for each breathing cycle	Implied 1:1	Implied 1:1	Implied 1:1	Implied 1:1
(6) Breathing intervention				
Number of laboratory sessions of the HRV-BF intervention	Pre- and post-operation	One	One	One
Minutes at the laboratory actually breathing (not doing other experimental tasks)	5 min practice, 10 min intervention	10 min	10 min	(2×) 10 min
Number of home-practice sessions (optional)	N/A	N/A	N/A	N/A
Minutes per each home-practice session (optional)	N/A	N/A	N/A	N/A
Control assessment for the home-practice sessions (optional)	N/A	N/A	N/A	N/A
(7) Conditions of the intervention				
Laboratory conditions: ambient light	N/A	Reduced distraction for screen-based experience	N/A	N/A
Laboratory conditions: temperature of the room	N/A	N/A	N/A	N/A
Laboratory conditions: body position	Reclined	Seated	Seated	Seated
Laboratory conditions: time of day	N/A	N/A	N/A	N/A
Laboratory conditions: number of participants at the same time	1	N/A	N/A	Small groups
Laboratory conditions (optional): smell/aroma, humidity, and eyes (closed or open)	N/A	N/A	N/A	N/A
Home-practice sessions (optional): such as time of day and ambient conditions	N/A	N/A	N/A	N/A
Recommendations for participants: such as caffeine or alcohol intake, minimum hours of sleep, and exercise before the session	N/A (pre-surgery recommendations)	Detailed	N/A	Detailed
Control of the recommendations for participants: how to assess the accomplishment of the previous recommendations and what to do if a participant does not follow them	N/A	N/A	N/A	N/A
(8) Equipment				
Biofeedback apparatus: brand, type of metronome, is it validated? Is it invasive? etc.	Custom, not validated	Custom, not validated	Custom, not validated	Custom, not validated
HRV apparatus (optional): brand, metronome, and biofeedback. Is it validated, or is it invasive? etc., for the analysis of heart rate variability	BN-ECG and BN-PPGED on hands	Polar H7 chest strap	Polar H7 chest strap	Polar H10 chest strap
Other apparatus for physiological analysis that can interact with the breathing performance, such as a thoracic belt for breathing analysis (e.g., respirometer)	Biopac BioNomadix BNRESP respiration band	N/A	N/A	N/A

BPM, breaths per minute; HRV-BF, heart rate variability biofeedback; N/A, not available; TKA, total knee arthroplasty.

(Left Column) Checklist with Eight Areas for Consideration and Reporting: (1) Registration, (2) Sample, (3) Allocation, (4) Missing Participants and Missing Data, (5) Breathing Protocol, (6) Breathing Intervention, (7) Conditions of the Intervention, (8) and Equipment.^9^ (Right Columns) Details Reported for Each Reviewed Study.
